# Зависимость течения болезни Иценко–Кушинга и результатов радикального лечения от МР-картины гипофиза у детей

**DOI:** 10.14341/probl12854

**Published:** 2022-04-12

**Authors:** Э. А. Янар, Н. В. Маказан, М. А. Карева, А. В. Воронцов, В. П. Владимирова, О. Б. Безлепкина, В. А. Петеркова

**Affiliations:** Национальный медицинский исследовательский центр эндокринологии; Национальный медицинский исследовательский центр эндокринологии; Национальный медицинский исследовательский центр эндокринологии; Национальный медицинский исследовательский центр эндокринологии; Национальный медицинский исследовательский центр эндокринологии; Национальный медицинский исследовательский центр эндокринологии; Национальный медицинский исследовательский центр эндокринологии

**Keywords:** синдром Иценко-Кушинга, АКТГ-секретирующая аденома, транссфеноидальная аденомэктомия, лучевая терапия, радиохиругия, ремиссия, рецидив, предиктор, дети

## Abstract

**ОБОСНОВАНИЕ:**

ОБОСНОВАНИЕ. Болезнь Иценко-Кушинга (БИК) – заболевание, причиной которого является гиперпродукция кортизола надпочечниками вследствие стимуляции АКТГ-секретирующей аденомой гипофиза (кортикотропиномой). Первым этапом терапии является хирургический метод лечения - аденомэктомия, который в 70-90% случаев приводит к ремиссии заболевания. Но даже при достижении ремиссии до 25% пациентов развивают рецидив заболевания. При неэффективности хирургического метода лечения или после развития рецидива возможно проведение лучевой терапии, которая также в 90% случаев приводит к ремиссии, но при которой чаще отмечаются осложнения в виде гипопитуитаризма.

**ЦЕЛЬ:**

ЦЕЛЬ. Анализ течения БИК и результатов лечения в зависимости от визуализации аденомы при проведении МРТ гипофиза и применяемых методов лечения в педиатрической практике.

**МАТЕРИАЛЫ И МЕТОДЫ:**

МАТЕРИАЛЫ И МЕТОДЫ. Ретроспективное исследование течения БИК у 91 ребенка, наблюдавшихся в период с 1992г по 2020г.

**РЕЗУЛЬТАТЫ:**

РЕЗУЛЬТАТЫ. По данным обследования были выявлены признаки аденомы гипофиза у 59% пациентов [54/91], неоднородность гипофиза - у 41% пациентов [37/91]. В 69% случаев [63/91] было проведено оперативное удаление аденомы гипофиза, в 31% – лучевая терапия [28/91]. После проведенного оперативного лечения ремиссия заболевания была достигнута у 71% пациентов [45/63], после проведенной лучевой терапии ремиссия была установлена у 82% пациентов [23/28]. Статистически значимых различий в достижении ремиссии заболевания после радикального лечения в зависимости от МРТ-характеристик выявлено не было (Р=0,21 после хирургического лечения и Р=0,83 после лучевой терапии, тест Хи-квадрат). Развитие рецидива было зафиксировано у 11 пациентов. Связи МРТ-характеристик со сроком развития рецидива выявлено не было (р=0,055, тест Хи-квадрат). Сроки рецидива статистически значимо различались у пациентов после проведенного хирургического лечения по сравнению с лучевой терапией (р=0,022, лог-ранговый тест) и в группе пациентов с выявленным гипокортицизмом в раннем послеоперационном периоде (р=0,04, лог-ранговый тест). При анализе эндокринных осложнений радикального лечения статистически значимых различий в частоте развития гипопитуитаризма в зависимости от размера кортикотропином не выявлено (р>0,002 после поправки Бонферрони, метод Хи-квадрат), однако получено статистически значимо более частое развитие всех компонентов гипопитуитаризма у пациентов после проведенной лучевой терапии (р<0,002 после поправки Бонферрони, метод Краскела-Уоллиса)

**ЗАКЛЮЧЕНИЕ:**

ЗАКЛЮЧЕНИЕ. Полученные результаты не позволяют использовать МРТ-характеристики кортикотропиномы, как предиктор эффективности терапии у пациентов с БИК в детской популяции. Выбор метода лечения влияет только на сроки развития рецидива заболевания, но не на его вероятность. Подтверждено статистически более частое развитие всех компонентов гипопитуитаризма после проведения лучевой терапии по сравнению с хирургическим лечением.

## Abstract (en)

**BACKGROUND:**

BACKGROUND: Cushing’s disease (CD) is a rare disorder of a persistent cortisol excess caused by ACTH-secreting pituitary tumor (corticotropinoma). Transsphenoidal surgery (TSS) is a treatment of choice for СD, which effectiveness range is from 70 to 90%. Recurrence rate after successful treatment is about 25%. If surgical treatment is unsuccessful or recurrence appear, radiation treatment is the next therapeutic option, which effectiveness range is also 90%, but the hypopituitarism rate as side effect of treatment is higher. Preoperative predictors of remission and recurrence are still unexplored what leads to further investigations.

**AIM:**

AIM: Analysis of remission and recurrence rates of pediatric CD after successful treatment according to preoperative MRI and therapeutic option.

**MATERIALS AND METHODS:**

MATERIALS AND METHODS: We conducted a retrospective analysis of 90 pediatric patients with CD who were observed between 1992 and 2020 at the Endocrinology Research Centre.

**RESULTS:**

RESULTS: The most common clinical symptoms of CD were weight gain [94%] and growth retardation [72%]. Pituitary tumor was detected on radiological imaging in 53/90 patients [59%], there were no signs of visible adenoma in 37/90 of patients [41%]. 63 of 90 patients underwent TSS (70%), 27 patients underwent radiosurgery (30%). Remission rate after TSS was 71% [45/63], after radiosurgery — 85% [23/27]. There were no significant differences in remission rates after radical treatment according to preoperative MRI results (P=0.21 after TSS and P=0.87 after radiosurgery, х2 analysis). Recurrence after successful treatment was diagnosed in 10 patients. There were no significant differences in time to recurrence according to preoperative MRI results (P=0.055, х2 analysis). Time to recurrence was statistically different after TSS compared to radiosurgery (P=0.007, Kaplan–Meier analysis) and in the group with developed adrenal insufficiency in the early postoperative period (P=0.04, Kaplan–Meier analysis). Analysis of side effect of treatment showed that the frequency of growth hormone and gonadotrophin deficiency was statistically higher after radiosurgery (р<0.01, Kruskel–Wallis ANOVA test). Diabetes insipidus was diagnosed only after TSS. CONCLUSION: Results of our study didn`t allow to use MRI-results as predictor of effectiveness treatment in pediatric CD. Therapeutic option has an impact on time to recurrence, not on recurrence rates. The frequency of growth hormone and gonadotrophin deficiency was statistically higher after radiosurgery compared to TSS. Further studies are needed to identify predictors of remission and recurrence in CD.>< 0.01, Kruskel–Wallis ANOVA test). Diabetes insipidus was diagnosed only after TSS.

## ОБОСНОВАНИЕ

Эндогенный гиперкортицизм — редкое мультисистемное заболевание, причиной которого является хроническая гиперпродукция гормона коры надпочечников — кортизола. Частота возникновения новых случаев эндогенного гиперкортицизма — 0,7–2,4 на 1 млн людей ежегодно, из них только 10% случаев встречается в педиатрической практике [1][2]. Генез гиперкортицизма может быть АКТГ-зависимым (АКТГ-продуцирующая аденома гипофиза (кортикотропинома) и АКТГ-эктопированный синдром) и АКТГ-независимым (автономная продукция кортизола опухолью (кортикостерома) или узелковой гиперплазией надпочечников; АКТГ — адренокортикотропный гормон) [2].

Кортикотропинома является источником автономной гиперпродукции АКТГ и, как следствие, хронического гиперкортицизма и приводит к развитию болезни Иценко–Кушинга (БИК). Как и во взрослой популяции, у детей кортикотропинома является самой частой причиной эндогенного гиперкортицизма, составляя 75–80% случаев, и чаще встречается в возрасте от 6 лет и старше [2][3].

Наиболее часто кортикотропиномы являются микроаденомами, частота макроаденом не превышает 10–20% [4–6]. При выявлении кортикотропиномы первым этапом лечения является аденомэктомия, приводя к ремиссии, по разным источникам, от 70 до 90% случаев [7–11]. На показатели эффективности, помимо хирургической методики и квалификации хирурга, влияют визуализация аденомы, ее размер и инвазия в синусы [4][10–16]. Во взрослой популяции, даже при достижении ремиссии после удаления аденомы, частота рецидива заболевания достигает 25% [17][18]. При возникновении рецидива возможно проведение повторного оперативного лечения, эффективность которого снижается до 50–70% [6][8][19], или лучевого лечения, что в 90–95% случаев приводит к ремиссии заболевания [20–22].

На настоящий момент есть много проспективных и ретроспективных исследований результатов лечения кортикотропином во взрослой популяции, но в связи с редкой встречаемостью мало исследований у детей.

## ЦЕЛЬ ИССЛЕДОВАНИЯ

Анализ течения БИК в зависимости от размера аденомы гипофиза и применяемых методов лечения в педиатрической практике.

## МАТЕРИАЛЫ И МЕТОДЫ

Место и время проведения исследования

Место проведения. ФГБУ «Национальный медицинский исследовательский центр эндокринологии» Минздрава России, Москва, Россия.

Время исследования. В период с 1992 по 2020 г.

Изучаемые популяции (одна или несколько)

Популяция: одна.

Критерии включения: возраст на момент установления диагноза менее 18 лет; подтвержденный центральный генез эндогенного гиперкортицизма.

Критерии исключения: подтверждение другого генеза гиперкортицизма, отсутствие данных по динамическому наблюдению после проведенного радикального лечения.

Способ формирования выборки из изучаемой популяции (или нескольких выборок из нескольких изучаемых популяций)

Сплошной.

Дизайн исследования

Одноцентровое наблюдательное динамическое одновыборочное исследование с ретроспективным компонентом.

Описание медицинского вмешательства (для интервенционных исследований)

Для анализа использованы данные историй болезни пациентов. Все медицинские вмешательства проводились вне исследования в рамках рутинной клинической практики по актуальным на соответствующий момент времени международным стандартам и строго при наличии показаний у каждого конкретного пациента.

Методы

Диагноз эндогенного гиперкортицизма подтверждался на основании 2 положительных лабораторных тестов из 3: нарушенного ритма секреции кортизола (нормальный или высокий уровень кортизола утром (≥600 нмоль/л), высокий уровень кортизола вечером (>300 нмоль/л) и АКТГ (нормальный или высокий уровень АКТГ утром (≥60 пг/мл), высокий уровень АКТГ вечером(>25 пг/мл), повышенного уровня кортизола в суточной моче (>400 нмоль/сут), отсутствия подавления уровня кортизола после ночного теста/малой дексаметазоновой пробы (ночной тест — 1 мг на ночь/малый тест с дексаметазоном — 30 мг/кг, максимально 0,5 мг через каждые 6 ч = по 2 мг/сут в течение 48 ч, всего 4 мг) [2].

После подтверждения эндогенного гиперкортицизма проводилась большая дексаметазоновая проба (120 мг/кг массы тела, максимально 2 мг/кг каждые 6 ч, максимально по 8 мг/сут в течение 48 ч, всего 16 мг) для дифференциальной диагностики между кортикотропиномой и АКТГ-эктопированным синдромом. Подавление уровня кортизола более чем на 50% исходного рассматривалось как подтверждение центрального генеза АКТГ-зависимого гиперкортицизма [2].

Для топической диагностики проводилась МРТ головного мозга с контрастным усилением (гадолиний), до 2014 г. исследования проводились на высокопольном аппарате МРТ мощностью 1 Тесла, в дальнейшем — на высокопольном аппарате МРТ мощностью 1,5 Тесла. По данным исследований данная разница в мощности аппаратов не влияет на четкость изображения и выявляемость объемных образований ЦНС [23][24]. При отсутствии визуализации аденомы для дифференциальной диагностики проводился селективный забор крови из нижних каменистых синусов со стимуляцией десмопрессином. Градиенты АКТГ>2 между центром и периферией до стимуляции и >3 после стимуляции десмопрессином рассматривались в качестве свидетельства центрального генеза гиперкортицизма. В нашей группе пациентов данное исследование проведено 17 пациентам, у всех был подтвержден диагноз БИК.

Всем пациентам после установления диагноза проведено лечение: 1-й группе пациентов — трансназальная аденомэктомия, 2-й группе пациентов — лучевое лечение (протонотерапия/ радиохирургия на установке гамма-нож). Выбор метода лучевого лечения был обусловлен временем первичного обследования и существующими на тот момент клиническими рекомендациями. До 2006 г. у детей с БИК 1-й линией лечения была протонная терапия, с 2006 г. методом выбора является хирургическое лечение, при неэффективности или невозможности его проведения начали использовать стереотаксическую радиохирургию на установке гамма-нож. Протонную терапию получили 30 пациентов (27 пациентов в качестве 1-го этапа терапии, 3 пациента — 2-го этапа), а лечение на установке гамма-нож — 6 пациентов (5 пациентов в качестве 2-го этапа терапии). Доза облучения при протонотерапии составляла 40–85 Гр, 3 пациента получили повторный курс протонотерапии, 1 пациент по поводу рецидива (40 Гр), 2 пациента по поводу неэффективного 1-го курса лучевого лечения (ЛЛ) (40 и 34,5 Гр соответственно). При проведении радиохирургии доза облучения составила 20–35 Гр. Хирургическое лечение проводилось в ФГБУ «НМИЦ эндокринологии» и ФГАУ «НМИЦ нейрохирургии им. Бурденко», протонотерапия проводилась в НТЦ «Медицинская физика ИТЭФ», радиохирургия на установке гамма-нож проводилась в ФГАУ «НМИЦ нейрохирургии им. Бурденко» и ФГБУ «НМИЦ ДГОИ им. Дмитрия Рогачева» МЗ РФ.

Эффективность хирургического метода лечения (ХЛ) подтверждалась в раннем послеоперационном периоде при достижении нормализации уровня кортизола или развитии гипокортицизма (уровни кортизола менее 50 нмоль/л). У пациентов, получивших ЛЛ в качестве 1-го этапа терапии, эффективность оценивалась не раньше чем через 6 мес по нормализации или снижению уровня свободного кортизола в суточной моче (<400 нмоль/сут). Рецидив заболевания подтверждался при развитии клинико-лабораторной картины гиперкортицизма после подтвержденной эффективности лечения.

Статистический анализ

Статистическая обработка полученных данных проводилась с использованием пакета статистических программ Statistica 13. Распределения количественных признаков представлены медианами (Me) и интерквартильными интервалами [Q1; Q3], качественных признаков — абсолютными и относительными частотами, их 95% доверительными интервалами (ДИ). Для сравнения групп использовались метод Краскела–Уоллиса и критерий Манна–Уитни (для количественных признаков), точный критерий Фишера и Хи-квадрат (для качественных признаков), лог-ранговый тест (при анализе времени до события). Пороговым уровнем статистической значимости Р считали 0,05. Для нивелирования проблемы множественных сравнений применяли поправку Бонферрони.

Этическая экспертиза

Протокол исследования был одобрен на заседании локального этического комитета ФГБУ «НМИЦ эндокринологии» Минздрава России (протокол №18 от 24.10.2018 г.).

## РЕЗУЛЬТАТЫ

Пациенты

В исследование были включены 90 детей с БИК, наблюдавшихся в период с 1992 по 2020 г. в ФГБУ «НМИЦ эндокринологии» Минздрава России. Все пациенты были разделены на 3 группы в зависимости от результатов МРТ гипофиза: 1-я группа — пациенты с невизуализируемой аденомой (n=37), 2-я группа — пациенты с микроаденомой гипофиза (<10 мм; n=40), 3-я группа — пациенты с макроаденомой гипофиза (>10 мм; n=13) (табл 1). Средний возраст манифестации первых симптомов гиперкортицизма в группах составил 10–11 лет, тогда как на момент установления диагноза средний возраст колебался от 13,5 до 15,5 года. При сравнении данных показателей по группам статистически значимых различий выявлено не было (табл. 2). Наиболее частыми клиническими проявлениями среди описанной группы пациентов являлись снижение темпов роста в совокупности с прогрессирующей прибавкой массы тела и перераспределением подкожно-жировой клетчатки по «кушингоидному» типу (табл. 1).

Статистически значимых различий в группах по показателям роста, скорости роста и веса также выявлено не было (см. табл. 2).

Реже встречались такие проявления хронического гиперкортицизма, как головные боли, артериальная гипертензия, различные нарушения углеводного обмена, гипокалиемия и остеопороз (см. табл. 1). Статистически значимой разницы в частоте развития данных осложнений гиперкортицизма в зависимости от размера аденом выявлено не было (см. табл. 2). Был проведен анализ ассоциации развития остеопороза с отставанием в росте, статистически значимых различий выявлено не было (р=0,77; точный критерий Фишера). Был проведен анализ гормонального профиля пациентов (базальные уровни АКТГ, кортизола, кортизола в суточном анализе мочи), статистически значимых различий этих показателей в зависимости от размера аденомы выявлено не было (см. табл. 2).

У пациентов с БИК с отставанием в росте (n=65) среднее время до установления диагноза составило 3,46 года (Q1: 2,1–Q3: 4,9 года), а у пациентов без отставания в росте (n=24) — 2,5 года (Q1: 0,9–Q3: 2,9 года), различия статистически значимы (р=0,006, критерий Манна–Уитни). Анализ частоты развития других проявлений гиперкортицизма в зависимости от длительности гиперкортицизма не выявил статистически значимых различий (табл. 3). Отмечена корреляция между степенью задержки роста и сроком от момента манифестации до диагностики заболевания. При этом не выявлено ассоциации между длительностью течения заболевания и остеопорозом.

**Table:** Таблица 1. Характеристика группы пациентов

Признак	Количество пациентов (n=90)
Пол
Женский, n (%)	46 (51)
Мужской, n (%)	44 (49)
Клинические проявления
Перераспределение подкожножировой клетчатки, n (%)	85 (94)
Замедление темпов роста, n (%)	65 (72)
Головная боль, n (%)	40 (44)
Артериальная гипертензия, n (%)	43 (48)
Нарушения углеводного обмена, n (%)	8 (9)
Остеопороз, n (%)	17 (19)
Гипокалиемия, n (%)	4 (4)
Размеры аденомы
Микроаденома, n (%)	40 (45)
Макроаденома, n (%)	13 (14)
Диффузная неоднородность гипофиза, n (%)	37 (41)

**Table:** Таблица 2. Клинико-лабораторные особенности групп пациентов, разделенных в зависимости от МРТ-характеристик аденомы гипофиза

Параметр	Группа 1 (неоднородность гипофиза)N=37	Группа 2 (микроаденома)N=40	Группа 3(макроаденома)N=13	Р
Средний возраст манифестации, годы,Me [Q1; Q3]	10 [ 8; 12]	11 [ 8,1; 13]N=39	10 [ 7; 12]	0,60
Средний возраст на момент установления диагноза, годыMe [Q1; Q3]	13,5 [ 11,45; 15,5]	14 [ 11,15; 15,5]	15,5 [ 14,3; 16,8]	0,088
Среднее время до установления диагноза, годы Me [Q1; Q3]	3 [ 2; 3]	3 [ 2; 3]N=39	4,3 [ 2,9; 5,7]	0,059
SDS ИМТ, Me [Q1; Q3]	2,145 [ 1,4; 3,1]N=32	2,345 [ 1,83; 2,92]N=30	2,17 [ 1,7; 3,3]N=11	0,86
SDS роста, Me [Q1; Q3]	-1,89 [ -3,15; -0,79]N=32	-1,74 [ -2,93; -0,4]N=30	-1,2 [-2,2; -0,13]N=11	0,37
Скорость роста, см/год, Me [Q1; Q3]	1,87 [ 0,8; 2,49]N=11	1,04 [ 1; 1,98]N=5	0,3 [ 0,3; 0,3]N=1	0,26
SDS скорости роста, Me [Q1; Q3]	-5,1 [ -5,6; -1,98]N=11	-2,5 [-3; -2,43]N=5	-5,74 [-5,7; -5,7] N=1	0,53
АКТГ (утро), пг/мл, Me [Q1; Q3]	51 [ 36,7; 67]N=29	55,1 [ 33,5; 87]N=33	85 [ 63,6; 99]N=13	0,16
Кортизол (утро), нмоль/л, Me [Q1; Q3]	759,2 [ 535,5; 1069,5]N=36	682 [ 613; 827]N=37	940 [ 759; 996]N=13	0,29
АКТГ (вечер), пг/мл, Me [Q1; Q3]	45,6 [ 23,6; 76]N=26	58 [ 45; 78,5]N=29	78 [ 50; 92]N=11	0,11
Кортизол (вечер), нмоль/л, Me [Q1; Q3]	681,1 [ 530; 955]N=31	660 [ 516,5; 789]N=35	743 [ 414; 928]N=11	0,66
Кортизол (суточная моча), нмоль/сут, Me [Q1; Q3]	1304 [ 868; 2475,2]N=34	1790 [ 1034; 2530]N=31	1663,9 [ 690; 4025]N=8	0,67
Артериальная гипертензия,n (%), 95% ДИ	18 (48)[ 32–65]	18 (46)[ 30–63]N=39	7 (54)[ 25–81]	0,89
Остеопороз,n (%), 95% ДИ	8 (22)(10–38)	7 (18)(7–33)N=39	2 (15)(2–45)	0,86
Нарушение толерантности к углеводам, n (%), 95% ДИ	3 (8)(2–22)	3 (8)(2–21)N=39	2 (15)(2–45)	0,68
Гипокалиемия,n (%), 95% ДИ	2 (5)(0–18)	1 (3)0–13)N=39	1 (8)(0–36)	0,70

**Table:** Таблица 3. Анализ зависимости частоты развития осложнений от длительности гиперкортицизма

Осложнение гиперкортицизма	Р
Отставание в росте	0,006
Артериальная гипертензия	0,78
Остеопороз	0,32
Нарушение толерантности к углеводам	0,22
Гипокалиемия	0,23

После подтверждения диагноза АКТГ-зависимого гиперкортицизма все пациенты получили лечение по поводу кортикотропиномы: у 70% пациентов (63/90) 1-м этапом лечения было ХЛ, а у оставшихся 30% пациентов (27/90) — ЛЛ.

После первоначально проведенного ХЛ ремиссия заболевания была достигнута у 71% пациентов (45/63), в группе с неоднородностью гипофиза — 63% (12 /19), в группе микроаденом (23/32) — 72%, макроаденом — 83% (10/12). Статистически значимых различий в частоте развития ремиссии заболевания после ХЛ в зависимости от визуализации аденомы по результатам МРТ получено не было (Р=0,21, тест Хи-квадрат) (табл. 4).

**Table:** Таблица 4. Ремиссия после проведенного 1-го этапа лечения

Вид лечения/результат	Все пациенты	Диффузная неоднородность	Микроаденомы	Макроаденомы	Р
1-й этап ХЛ	63	19	32	12	
Ремиссия, n (%)95% ДИ	45 (71)(58–82)	12 (63)(38–84)	23 (72)(53–86)	10 (83)(52–98)	0,21
1-й этап ЛЛ	27	18	8	1	
Ремиссия, n (%)95% ДИ	23 (85)(66–96)	15 (83)(58–96)	7 (87)(47–99)	1 (100)(25–100)	0,87

Из 27 пациентов, получивших ЛЛ, ремиссия была установлена у 85% пациентов (23/27): из них в группе с неоднородностью — 83% (15/18), в группе микроаденом эффективность составила 87% (7/8), в группе макроаденом — 100% (1/1). Статистически значимых различий в частоте развития ремиссии заболевания после ЛЛ в зависимости от визуализации аденомы по результатам МРТ получено не было (Р=0,87, тест Хи-квадрат) (см. табл. 4).

Сравнение трех независимых групп проводилось при помощи теста Хи-квадрат. Пороговый P=0,002 (после применения поправки Бонферрони).

У 11 (61%) пациентов из 18 с доказанной неэффективностью 1-го этапа терапии (ХЛ) было проведено повторное удаление аденомы гипофиза, из них в 73% случаев (8/11) была достигнута ремиссия заболевания. Три пациента, у которых не была достигнута ремиссия заболевания, получили ЛЛ, у одного пациента достигнута ремиссия заболевания, у двух пациентов не удалось оценить эффект ЛЛ в связи с коротким периодом наблюдения после его проведения.

6 пациентов из 18 после неэффективности 1-го этапа ХЛ 2-м этапом терапии получили ЛЛ (33%), в 100% случаев была достигнута ремиссия заболевания (6/6). Статистически значимых различий в частоте развития ремиссии заболевания после 2-го этапа ХЛ в зависимости от визуализации аденомы по результатам МРТ получено не было (Р=0,3; тест Хи-квадрат).

У 4 (15%) пациентов из 27 проведенное на 1-м этапе ЛЛ оказалось неэффективным. Среди них 3 пациентам был проведен повторный сеанс ЛЛ и у всех пациентов достигнута ремиссия. Одному пациенту после неэффективного ЛЛ в качестве 2-го этапа было проведено ХЛ, достигнута ремиссия заболевания. Сравнение групп не смогло быть вычислено в связи с малым количеством частот.

При достижении ремиссии заболевания после радикального лечения БИК проводилось динамическое наблюдение пациентов. Рецидив заболевания подтверждался при развитии клинико-лабораторной картины гиперкортицизма после достижения ремиссии заболевания. Данные обо всех пациентах, развивших рецидив заболевания, представлены в табл. 6.

Был проведен анализ сроков развития рецидива в зависимости от размеров аденом, проведенного лечения и развития гипокортицизма после ХЛ на 1-м этапе. Для всех изучаемых предикторов был проведен анализ времени до события методом Каплана–Майера.

Как было указано выше, пациенты были разделены на 3 подгруппы в зависимости от размера аденомы. Рецидив произошел у 2/37 пациентов с неоднородностью гипофиза (5% [0,6; 18]), у 5/40 пациентов с микроаденомой (12% [4; 27]) и у 3/13 пациентов с макроаденомой (23% [ 5; 54]). Различия частоты случаев рецидива в подгруппах статистически незначимы (р=0,055, тест Хи-квадрат, пороговый P=0,025 (после применения поправки Бонферрони)) (рис. 1), что указывает на отсутствие ассоциации МРТ-характеристик с вероятностью рецидива. Влияние инвазии на вероятность возникновения рецидива не оценивали в связи с малым количеством имеющихся данных.

Проведен анализ срока рецидива с проведенным лечением. Все пациенты (n=90) были разделены на 2 группы: 1-я группа — получившие только ХЛ (n=55), 2-я группа — получившие на одном из этапов ЛЛ (n=35). В 1-й группе пациентов рецидив произошел у 8/55 пациентов (14% [ 6; 26]), а во 2-й группе — у 2/35 пациентов (6% [ 1; 19]). Сроки рецидива статистически значимо различались у пациентов после проведенного ХЛ (средний срок — 3,3 года) по сравнению с ЛЛ (средний срок 6 лет) (р=0,007, лог-ранговый тест, пороговый P=0,025 (после применения поправки Бонферрони); рис. 2).

Проведен анализ сроков возникновения рецидива в зависимости от развития гипокортицизма после проведенного ХЛ (кортизол менее 50 нмоль/л в раннем послеоперационном периоде), что на настоящий момент является одним из самых эффективных предикторов длительной ремиссии заболевания по данным литературы. Все пациенты, получившие первым этапом ХЛ (n=63), были разделены на 2 группы: 1-я группа — без подтвержденного гипокортицизма (n=23), 2-я группа — с подтвержденным гипокортицизмом в раннем послеоперационном периоде (n=38), у 2 пациентов не было получено данных об уровне послеоперационного кортизола. В 1-й группе пациентов рецидив произошел у 4/23 пациентов (17% [ 5; 39]), а во 2-й группе — у 5/38 пациентов (13% [ 4; 28]). Сроки рецидива статистически значимо различались у пациентов с послеоперационным гипокортицизмом, рецидив заболевания в этой группе пациентов возникал в более ранние сроки по сравнению с пациентами без достигнутого гипокортицизма в раннем послеоперационном периоде (р=0,04, лог-ранговый тест, пороговый P=0,05) (рис. 3).

**Figure:**
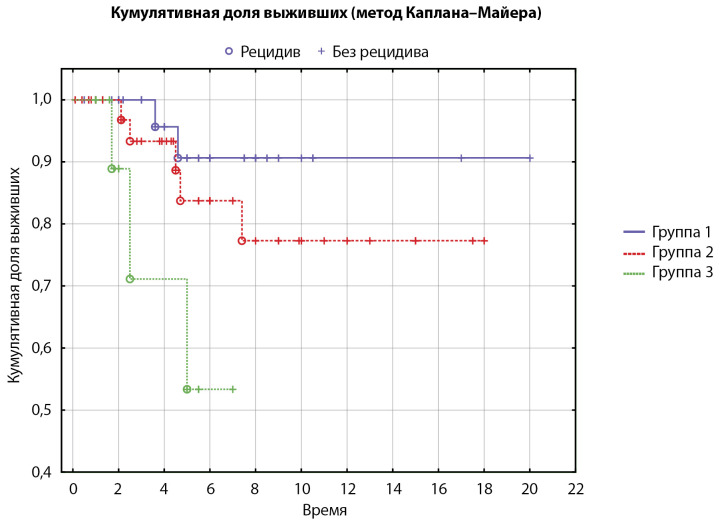
Рисунок 1. Время до рецидива в подгруппах, сформированных в зависимости от МРТ-характеристик аденом гипофиза (кривые Каплана–Майера, n=90). Группа 1 — пациенты с неоднородностью гипофиза (n=37, рецидив у 2 пациентов), группа 2 — с микроаденомой гипофиза (n=40, рецидив у 5 пациентов), группа 3 — с макроаденомой гипофиза (n=13, рецидив у 3 пациентов). Среднее время до наступления рецидива: 1-я группа — 4,1 года, 2-я группа — 4,1 года, 3-я группа — 3 года.

**Figure:**
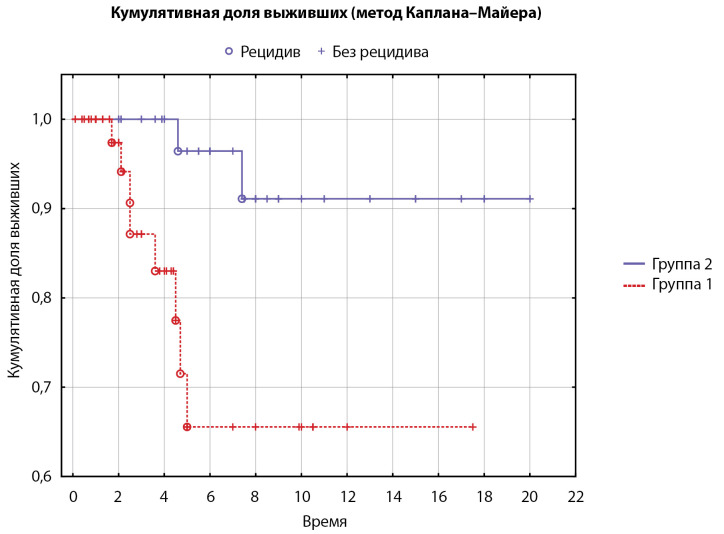
Рисунок 2. Время до рецидива в подгруппах, сформированных в зависимости от метода лечения (кривые Каплана–Майера, n=90). Группа 1 — пациенты после хирургического лечения (n=55, рецидив у 8 пациентов), группа 2 — после лучевого лечения на одном из этапов (n=36, рецидив у 2 пациентов). Среднее время до наступления рецидива: 1-я группа — 3,3 года, 2-я группа — 6 лет.

**Figure:**
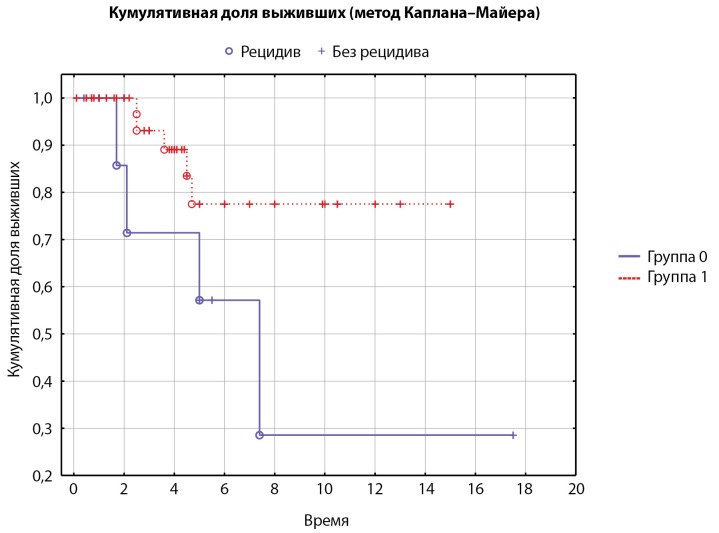
Рисунок 3. Время до рецидива в подгруппах, сформированных в зависимости от развития гипокортицизма после радикального лечения (кривые Каплана–Майера, n=63). Группа 0 — пациенты, не развившие гипокортицизм (n=23, рецидив у 4 пациентов), группа 1 — развившие транзиторный или стойкий гипокортицизм (n=38, рецидив у 5 пациентов). Среднее время до наступления рецидива: 0-я группа — 3,56 года, 1-я группа — 2,65 года.

Проведен анализ развития эндокринных осложнений после эффективного лечения в зависимости от метода. Как было указано выше, пациенты были разделены на 2 подгруппы (группа 1 — пациенты после ХЛ, группа 2 — после ЛЛ). Дефицит соматотропного гормона (СТГ) развился у 49% пациентов (27/55) в 1-й группе против 91% (30/33) во 2-й. Вторичный гипотиреоз развился в 45% случаев (25/55) в 1-й группе против 61% (20/33) во 2-й. Вторичный гипогонадизм диагностирован у 27% пациентов (13/55) в 1-й группе против 48% (16/33) во 2-й. Несахарный диабет развился только в 1-й группе пациентов в 18% случаев (10/55). Частота развития СТГ-дефицита и вторичного гипогонадизма статистически значимо выше у пациентов после проведенной ЛЛ, статистически значимых различий в развитии вторичного гипотиреоза не выявлено (табл. 7). Несахарный диабет развивался у пациентов только после перенесенного ХЛ.

Сравнение трех независимых групп проводилось при помощи метода Краскела–Уоллиса. Пороговый Р=0,01 (после применения поправки Бонферрони).

**Table:** Таблица 6. Характеристика пациентов, развивших рецидив БИК

№	Пол	Возраст манифестации, годы	Возрастустановления диагноза, годы	Аденомапо данным МРТ	Метод лечения до рецидива	Гипокортицизм в исходе лечения	Время до наступления рецидива, годы	Метод лечения, после рецидива	Гипокортицизм в исходе
1	ж	14,10	14,90	Микроаденома	ХЛ	нет	2,11	ЛЛ	
2	м	10,00	14,30	Макроаденома	ХЛ	нет	1,70	ЛЛ	нет
3	м	8,00	13,60	Макроаденома	ХЛ	нет	5,00	ХЛ	да
4	ж	8,00	11,45	Не визуализировалась	ЛЛ+ЛЛ	нет	4,60	ЛЛ	нет
5	ж	8,00	15,50	Микроаденома	ХЛ	да	4,70	ХЛ	
6	ж	13,00	15,70	Микроаденома	ХЛ	да	2,50	ХЛ	нет
7	ж	13,00	15,50	Микроаденома	ХЛ	да	4,50	ХЛ	
8	ж	7,00	15,70	Макроаденома	ХЛ	да	2,50	ХЛ	да
9	ж	6,00	6,11	Не визуализировалась	ХЛ	да	3,60	ЛЛ	нет
10	ж	6,00	6,90	Микроаденома	ХЛ+ЛЛ	нет	7,40	ЛЛ	нет

**Table:** Таблица 7. Гипопитуитаризм в зависимости от проведенного лечения

Гипопитуитаризм	Все пациенты	После ХЛ	После ЛЛ	Р
Тропная недостаточность	88	55	33	
СТГ-дефицит, n (%)	57 (65)(53–75)	27 (49)(35–63)	30 (91)(76–98)	0,00007
Вторичный гипотиреоз, n (%)	45 (51)(40–62)	25 (45)(32–59)	20 (61)(42–77)	0,17
Вторичный гипогонадизм, n (%)	29 (33)(23–44)	13 (27)(16–41)	16 (48)(31–66)	0,01
Несахарный диабет, n (%)	10 (11%)(6–20)	10 (18%)(9–31)	0	0,009

## ОБСУЖДЕНИЕ

БИК — крайне редкая патология в детской популяции, что и обуславливает достаточно скудное количество исследований и небольшие группы пациентов.

Самым главным клиническим отличием течения гиперкортицизма у детей от взрослых является замедление темпов роста в совокупности с прибавкой массы тела, которые являются в большинстве случаев самыми первыми и частыми проявлениями манифестации заболевания. Другие клинические проявления в виде развития гирсутизма, различных нарушений полового созревания, головной боли, артериальной гипертензии, нарушений углеводного обмена и остеопороза встречаются гораздо реже [2][25]. Появление свойственных для гиперкортицизма стрий также не характерно для детей младше 6–7 лет [2]. В нашей группе пациентов частота клинических проявлений гиперкортицизма совпала с данными литературы. Более чем в 60% случаев была выявлена задержка роста, а жалобы на прогрессирующую прибавку массы тела предъявляли более 80% пациентов. Остальные клинические проявления встречались с частотой от 10 до 40% (см. табл. 1). По нашим данным, задержка роста не приводит к сокращению сроков постановки диагноза гиперкортицизма. Однако скорость роста оказалась единственным параметром, коррелирующим с длительностью течения гиперкортицизма до момента постановки диагноза (средний срок — 3,46 года), в отличие от формирования артериальной гипертензии, нарушений углеводного обмена и развития остеопороза.

По данным литературы, во взрослой и детской популяции наиболее часто размеры кортикотропином не превышают 10 мм (микроаденомы), частота выявления макроаденом не превышает 10–20% [4–6]. Отличием детской популяции также является высокий процент случаев отсутствия визуализации аденомы гипофиза, по некоторым данным, до 50% случаев по результатам МРТ описывается неизмененный или диффузно неоднородный гипофиз [25–27]. Полученные данные в нашей группе пациентов также совпадают с данными литературы: у 45% пациентов (41/91) была выявлена микроаденома гипофиза (<10 мм), у 14% пациентов (13/91) размеры аденомы превышали 10 мм (макроаденома), у 41% пациентов (37/91) признаков аденомы обнаружено не было. Различий в клинико-лабораторных характеристиках гиперкортицизма в зависимости от размера аденомы выявлено не было (см. табл. 2).

При установлении диагноза БИК необходимо максимально быстрое и радикальное лечение до возникновения тяжелых осложнений персистирующего гиперкортицизма. Золотым стандартом лечения на настоящий момент является аденомэктомия, так как есть возможность трансназального доступа с вероятностью изолированной аденомэктомии с сохранением нормальной ткани гипофиза и быстрой возможностью оценки эффекта терапии. Трансназальная аденомэктомия приводит к ремиссии во взрослой и детской популяции, по разным источникам, от 70 до 90% случаев [7–11][25–28]. В нашей группе пациентов ремиссия после ХЛ была достигнута в 73% случаев, что совпадает с данными литературы.

При отсутствии ремиссии после ХЛ или возникновении рецидива возможно проведение повторного оперативного лечения, эффективность которого снижается, по данным различных источников, до 50–70% [6, 8, 19, 25, 28]. В нашей группе пациентов после повторной аденомэктомии ремиссия гиперкортицизма была достигнута у 80% пациентов (8/10). При неэффективности повторного ХЛ или нецелесообразности/невозможности его проведения прибегают к ЛЛ, в 90–95% случаев приводящему к длительной ремиссии заболевания [20–22][28][29]. В нашем исследовании в связи с тем, что до 2006 г. протонная терапия являлась методом выбора лечения при отсутствии визуализации аденомы, пациенты, получившие ЛЛ, разделились на 2 подгруппы: 1-я — получившие ЛЛ на 1-м этапе лечения и 2-я подгруппа — получившие ЛЛ 2-м этапом после неэффективности ХЛ. Ремиссия была достигнута в 82% случаев в 1-й подгруппе (23/28) и в 100% случаев во 2-й подгруппе (6/6). Статистически значимых различий в частоте достижения ремиссии заболевания в зависимости от размера аденомы выявлено не было (см. табл. 4 и 5).

При достижении ремиссии во взрослой популяции частота рецидивов заболевания может достигать 25% [17][18]. В связи с маленькими выборками при исследованиях БИК у детей частота рецидивов колеблется от 0 до 20% [25–28]. В нашей группе пациентов общая частота рецидива составила 12% (11/91), после ХЛ рецидив был зафиксирован в 14% случаев (8/55), после ЛЛ — в 8% (3/36).

В связи с высокой частотой развития рецидива и невозможностью достижения ремиссии заболевания ведутся активный поиск и анализ предикторов развития рецидива и ремиссии.

Влияние размера аденом гипофиза на длительность ремиссии и вероятность развития рецидива достаточно неоднозначно. В ряде ретроспективных исследований у пациентов с микроаденомами в большем проценте случаев достигалась ремиссия заболевания, тогда как у пациентов с макроаденомой чаще не удавалось достигнуть ремиссии и развивался рецидив [14][30][31]. Противоположные данные были получены в ряде других исследований, где частота достижения ремиссии у пациентов с микроаденомами не отличилась от пациентов с макроаденомами [11][32][33].

Было предположено, что отсутствие визуализации аденомы по данным МРТ является неблагоприятным признаком, однако, по данным ряда исследований, не было выявлено связи между визуализацией аденомы по данным МРТ и частотой достижения ремиссии и возникновения рецидива после аденомэктомии [4][6][8][34].

По данным литературы, ряд исследователей отмечают зависимость частоты наступления ремиссии не столько от размера, сколько от наличия инвазивного роста опухоли, однако эти данные противоречивы. По некоторым данным, инвазивный рост опухоли являлся важнейшим фактором, влияющим на вероятность наступления ремиссии [11][13–16]. При этом в ряде других исследований такой зависимости обнаружено не было [4, 6].

По результатам R. Lonser et al. предикторами длительной ремиссии у детей являлись маленький размер аденомы с отсутствием инвазии [25]. Также были получены данные, совпадающие с работами ряда других исследователей, что утренние уровни кортизола и АКТГ в раннем послеоперационном периоде являются на данный момент самыми важными факторами, ассоциированными с развитием рецидива либо сохранением длительной ремиссии [31, 35, 36].

По результатам анализа данных, полученных на нашей группе пациентов, статистически значимых различий в достижении ремиссии заболевания и вероятности рецидива заболевания в зависимости от размера кортикотропином и метода лечения не выявлено (см. табл. 4 и 5). Влияние инвазии на вероятность возникновения рецидива не оценивали в связи с малым количеством имеющихся данных. Выявлена статистически значимая разница в сроках развития рецидива в зависимости от проведенного лечения, у пациентов после хирургического лечения отмечен рецидив заболевания в более ранние сроки по сравнению с пациентами, получившими лучевое лечение. Также выявлена связь между сроком развития рецидива гиперкортицизма и развитием надпочечниковой недостаточности после проведенного лечения. Среди пациентов с гипокортицизмом, развившимся после хирургического лечения, рецидив заболевания возникал в более ранние сроки по сравнению с пациентами, нормализовавшими уровень кортизола после операции. Можно предполагать, что развитие гипокортицизма приводит к более выраженной стимуляции гипоталамо-гипофизарной системы, что, возможно, ведет к стимуляции оставшихся опухолевых клеток, и следовательно, более раннему проявлению рецидива заболевания. Найденная корреляция не позволяет расценивать развитие гипокортицизма в послеоперационном периоде как предиктор риска рецидива заболевания.

При анализе эндокринных осложнений после проведенного лечения получено статистически значимое более частое развитие СТГ-дефицита и вторичного гипогонадизма у пациентов после проведенного ЛЛ по сравнению с ЛЛ. Несахарный диабет развивался только у пациентов после ХЛ (см. табл. 7).

## ОГРАНИЧЕНИЯ ИССЛЕДОВАНИЯ

Исследование является ретроспективным, поэтому нельзя исключить историческое смещение в оценке лабораторных показателей. С ретроспективным дизайном связано и значительное количество пропусков в данных, и выбор метода лечения, что оказывает влияние на течение и риски рецидива заболевания, осложнения после проведенного лечения. Мощность аппаратов МРТ (1 и 1,5 Тесла), различавшаяся в разные периоды времени исследования, не оказывала влияния на выявляемость объемных образований ГМ.

## ЗАКЛЮЧЕНИЕ

В связи с редкой встречаемостью БИК в детской популяции данные по анализу течения и риска развития рецидивов крайне малы. Согласно полученным результатам на большой группе пациентов, данные визуализации кортикотропином с помощью МРТ не являются предикторами достижения ремиссии и вероятности развития рецидива заболевания.

## ДОПОЛНИТЕЛЬНАЯ ИНФОРМАЦИЯ

Источник финансирования. Исследование выполнено в рамках государственного задания «Молекулярно-генетические, масс-спектрометрические и иммуногистохимические маркеры в персонализации диагностики и лечении гиперкортицизма у детей», регистрационный номер АААА-А20-120011790183-5

Конфликт интересов. Все авторы декларируют отсутствие явных и потенциальных конфликтов интересов, связанных с публикацией настоящей статьи.

Участие авторов. Все авторы одобрили финальную версию статьи перед публикацией, выразили согласие нести ответственность за все аспекты работы, подразумевающую надлежащее изучение и решение вопросов, связанных с точностью или добросовестностью любой части работы.
